# Could Incretin‐Based Weight Loss Unmask a Modifiable Nociplastic Pain Phenotype? A Rheumatological Reading of SURMOUNT‐5

**DOI:** 10.1111/dom.70936

**Published:** 2026-06-03

**Authors:** Jacopo Ciaffi, Roberto Caporali, Francesco Ursini

**Affiliations:** ^1^ Medicine and Rheumatology Unit IRCCS Istituto Ortopedico Rizzoli Bologna Italy; ^2^ Department of Biomedical and Neuromotor Sciences Alma Mater Studiorum University of Bologna Bologna Italy; ^3^ Department of Clinical Sciences and Community Health University of Milan Milan Italy; ^4^ Department of Rheumatology and Medical Sciences ASST Pini‐CTO Milan Italy

**Keywords:** antiobesity drug, GLP‐1, obesity therapy, pain, patient reported outcomes

1

We read with great interest the report by Shukla and colleagues on health‐related quality of life in SURMOUNT‐5, a head‐to‐head comparison of tirzepatide and semaglutide in adults with obesity or overweight without type 2 diabetes [1]. The authors show that both agents improved health‐related quality of life, with greater improvement in general health with tirzepatide. Of particular note, participants with baseline physical limitation experienced greater improvements in physical functioning, the physical component summary, and general health with tirzepatide than with semaglutide. In pooled analyses, increasing body‐weight reduction was associated with a graded improvement in physical functioning, role‐physical, bodily pain, general health and vitality, whereas the mental component summary did not follow the same pattern.

This symptom cluster may have an underappreciated rheumatological implication. The domains most responsive to greater weight loss (bodily pain, physical function and vitality) map closely onto the clinical burden of fibromyalgia and related nociplastic pain states. Fibromyalgia is no longer conceptualized as pain alone: contemporary criteria integrate widespread pain with symptom severity, including fatigue, unrefreshing sleep and cognitive symptoms [2]. European Alliance of Associations for Rheumatology (EULAR) recommendations similarly emphasize its heterogeneity and the centrality of non‐pharmacological strategies, particularly graduated physical activity, while acknowledging the frequent coexistence of fatigue, sleep disturbance, mood symptoms and impaired quality of life [3].

From this perspective, SURMOUNT‐5 may have captured, without explicitly naming it, a clinically meaningful pain‐function‐fatigue axis. The absence of parallel improvement in the mental component summary is important. This does not imply that psychological distress is irrelevant to nociplastic states, which frequently coexist with anxiety and depression, but rather suggests that the observed improvements may not be explained solely by mood‐ or body image‐related mechanisms. Instead, the data are compatible with a more specific hypothesis: substantial incretin‐based weight loss may expand physical capacity and reduce pain interference in a subgroup of patients whose symptom burden is amplified by obesity, deconditioning, sleep disruption, low‐grade inflammation, mechanical loading, adipokine dysregulation or altered nociceptive processing. Mechanical unloading and weight loss‐associated neuroimmune changes, including potential modulation of leptin‐related inflammatory pathways, likely contribute substantially to these effects [4, 5, 6]. Emerging evidence also suggests that microbiota‐immune interactions may contribute to obesity‐associated low‐grade inflammation and altered nociceptive processing, potentially representing an additional upstream driver of symptom amplification in nociplastic states [7]. Although these pathways remain incompletely characterized, they further support the biological complexity linking obesity, systemic inflammation and pain sensitization.

This broader conceptual framework is now supported by convergent preclinical and real‐world evidence. In a reserpine‐induced rat model of fibromyalgia, semaglutide improved hyperalgesia, motor incoordination and depressive‐like behaviour, while attenuating inflammatory changes in the spine and dorsal root ganglia [8]. Mechanistically, these effects were accompanied by activation of the cAMP/PKA/p‐CREB pathway and a shift in microglial/macrophage polarization away from a pro‐inflammatory M1 phenotype and toward an anti‐inflammatory M2 phenotype, with reduced TNF‐α and inducible nitric oxide synthase and increased IL‐4, arginase‐1 and CD163 expression [8]. Although animal models of fibromyalgia cannot be directly extrapolated to clinical disease, and the doses employed in preclinical studies may not correspond to those used for weight management in humans, these findings provide a plausible biological bridge between GLP‐1 receptor activation, neuroinflammation and nociplastic pain modulation. A complementary clinical signal has recently emerged from a large propensity‐matched TriNetX cohort of patients with fibromyalgia. After matching, 48,025 GLP‐1 receptor agonist users were compared with 48,025 non‐users. GLP‐1 receptor agonist use was associated with lower odds of opioid prescription, pain‐related diagnostic codes and fatigue‐related diagnostic codes, with odds ratios of 0.65, 0.79 and 0.68, respectively, while disability‐related codes did not differ significantly [9]. Importantly, BMI and HbA1c remained higher in the GLP‐1 receptor agonist cohort, suggesting that the observed associations cannot be dismissed simply as a marker of healthier metabolic status. Nevertheless, residual confounding, indication bias and reliance on diagnostic codes rather than validated fibromyalgia instruments remain important limitations.

Taken together, these data sharpen the question raised by SURMOUNT‐5. The relevant issue may not be whether incretin‐based therapies ‘treat fibromyalgia’ in a broad sense, but whether they improve a modifiable nociplastic symptom cluster in patients with obesity: bodily pain, low vitality, reduced activity tolerance and physical limitation. Obesity is associated with fibromyalgia prevalence and severity, and weight reduction has previously been associated with improvements in Fibromyalgia Impact Questionnaire scores, tender point count, depression and sleep quality [10, 11].

The language of nociplastic pain may be particularly useful here. Nociplastic pain describes pain arising from altered nociception without clear evidence of ongoing tissue damage or somatosensory lesion sufficient to explain the pain experience [12]. Fibromyalgia is commonly considered a prototypical nociplastic condition, although peripheral and small‐fibre abnormalities may contribute in subsets of patients [13]. This matters because obesity trials usually measure pain only indirectly, through generic instruments such as the SF‐36 bodily pain domain. Such instruments are valuable, but they cannot distinguish mechanical pain from widespread pain, pain interference, central sensitization, neuropathic‐like symptoms, or fibromyalgia‐spectrum illness.

We therefore suggest that future obesity pharmacotherapy trials incorporate a brief rheumatological phenotyping module. This could include the Widespread Pain Index and Symptom Severity Scale, the Fibromyalgia Impact Questionnaire–Revised, PROMIS Pain Interference, sleep quality and fatigue instruments, and a simple pain distribution body map. These measures would add little participant burden but would allow prespecified analyses of whether baseline widespread pain, fatigue, non‐restorative sleep, or pain interference modifies the quality‐of‐life response to tirzepatide or semaglutide. Such instruments could also serve as stratification variables to explore heterogeneity in pain and functional responses across obesity populations with differing nociplastic symptom burden. Mediation analyses could then evaluate whether improvements in bodily pain and vitality are explained primarily by percentage weight loss, waist circumference reduction, physical activity, sleep improvement, inflammatory biomarkers, glycemic change or treatment‐specific neuroimmune mechanisms.

Such work could be clinically consequential. In rheumatology practice, patients with fibromyalgia and obesity are often encouraged to increase physical activity, but pain, fatigue and post‐exertional symptom amplification may restrict the behavioural changes being prescribed [14]. This barrier may be particularly relevant in patients with nociplastic symptom profiles characterized by activity‐related symptom exacerbation and limited exercise tolerance. If incretin‐based obesity treatment increases the ‘activity envelope’ by improving pain, vitality and physical function, it could serve as an enabling intervention for guideline‐concordant rehabilitation. Conversely, if patients with a nociplastic phenotype experience attenuated benefit despite major weight loss, they may require earlier integrated care targeting sleep, pain education, pacing, psychological flexibility and graded activity.

We do not propose that SURMOUNT‐5 demonstrates efficacy in fibromyalgia. Fibromyalgia was not specifically phenotyped or reported in SURMOUNT‐5, nor was it listed among the published exclusion criteria; therefore, the prevalence of nociplastic symptom profiles within the trial population remains unknown. Rather, SURMOUNT‐5 generates a refined and now testable translational question: are the improvements in bodily pain, vitality and physical function after pharmacological weight loss evenly distributed across people with obesity, or enriched among those with an unrecognized nociplastic pain phenotype? The convergence of SURMOUNT‐5, preclinical semaglutide data, and real‐world fibromyalgia outcomes suggests that both a prespecified fibromyalgia/nociplastic‐pain substudy nested within future incretin trials and dedicated studies in rheumatological populations enriched for nociplastic symptoms would be timely and potentially high‐yield.

## Funding

The authors have nothing to report.

## Ethics Statement

The authors have nothing to report.

## Consent

The authors have nothing to report.

## Conflicts of Interest

Jacopo Ciaffi reports payment or honoraria for lectures, presentations or educational events from Amgen, IBSA and Novartis, and support for attending meetings and/or travel from AbbVie, Alfasigma and UCB. Francesco Ursini reports payment or honoraria for lectures, presentations or educational events from AbbVie, Novartis, Pfizer and Alfasigma, and support for attending meetings and/or travel from AbbVie, Novartis, Pfizer, Alfasigma and UCB. Roberto Caporali reports payment or honoraria for lectures, presentations or educational events from AbbVie, Alfasigma, Bristol Myers Squibb, Lilly, Novartis, UCB, GSK, AstraZeneca and Boehringer Ingelheim.

## Data Availability

No new data were generated or analysed for this commentary. All data discussed are derived from previously published studies cited in the manuscript.
